# The Current Approach to the Diagnosis and Classification of Mirizzi Syndrome

**DOI:** 10.3390/diagnostics11091660

**Published:** 2021-09-10

**Authors:** Jakub Klekowski, Aleksandra Piekarska, Marta Góral, Marta Kozula, Mariusz Chabowski

**Affiliations:** 1 Student Research Group No 180, Faculty of Medicine, Wroclaw Medical University, 50-367 Wrocław, Poland; klekowski.jakub@gmail.com (J.K.); aleksandra.piekarska@poczta.onet.pl (A.P.); marta.goral21@gmail.com (M.G.); marta.kozula@gmail.com (M.K.); 2 Division of Oncology and Palliative Care, Department of Clinical Nursing, Faculty of Health Science, Wroclaw Medical University, 5 Bartla Street, 51-618 Wrocław, Poland; 3 Department of Surgery, 4th Military Teaching Hospital, 5 Weigla Street, 50-981 Wrocław, Poland

**Keywords:** classification, ultrasonography (US), computed tomography (CT), magnetic resonance cholangiopancreatography (MRCP), endoscopic retrograde cholangiopancreatography (ERCP), preoperative diagnosis, cholecystobiliary fistula

## Abstract

Mirizzi syndrome occurs in up to 6% of patients with cholecystolithiasis. It is generally caused by external compression of the common hepatic duct by a gallstone impacted in the neck of the gallbladder or the cystic duct, which can lead to fistulisation. The aim of this review was to highlight the proposed classifications for Mirizzi syndrome (MS) and to provide an update on modern approaches to the diagnosis of this disease. We conducted research on various internet databases and the total number of records was 993, but after a gradual process of elimination our final review consisted of 21 articles. According to the literature, the Cesendes classification is the most commonly used, but many new suggestions have appeared. Our review shows that the ultrasonography (US) is the most frequently used method of initial diagnosis, despite still having only average sensitivity. Magnetic resonance cholangiopancreatography (MRCP) and endoscopic retrograde cholangiopancreatography (ERCP) are good methods and are similarly effective, but only the latter can be simultaneously therapeutic. Some modern methods show very high sensitivity, but are not so commonly administered. Mirizzi syndrome is still a diagnostic challenge, despite the advancement of the available tools. Preoperative diagnosis is crucial to avoid complications during treatment. New research may bring a unification of classifications and diagnostic algorithms.

## 1. Introduction

Mirizzi syndrome (MS) is a rare condition caused by the compression of the common hepatic duct due to stones located in the cystic duct or the neck of the gallbladder. The main symptoms noticed in patients with this condition are upper abdominal pain and jaundice [1,2,3]. It was first mentioned in 1905 by Kehr and later in 1908 by Ruge, who described it as a disease caused by the external obstruction of the bile duct associated with jaundice. Eventually, in 1948, the Argentinean surgeon Pablo Mirizzi defined it as the compression of a bile duct by a gallstone, associated with pressure ulceration generating local inflammation. The compression may lead to external obstruction, erosion, fibrosis or fistula with various levels of complexity [1,4,5]. It means that it can be generally depicted as an uncommon manifestation of cholelithiasis [2]. The reported frequency of MS is approximately 0.05–4%. Based on data presented in articles, the overall frequency of MS was higher in females than in males. The proportion of females suffering from MS ranged between 55.6–77% [6,7,8,9,10]. However, the available data vary in different parts of the world. Thus, in well-developed countries and regions, such as Europe, MS is found in 0.5% of all cholecystectomies, but in Asia, Central and South America the statistics are generally higher and reach as much as 4.7–5.7% [1,11,12,13,14,15]. In the population of patients undergoing endoscopic retrograde cholepancreatography (ERCP), the incidence of MS is estimated to be 1.07% [5].

**Figure 1:**
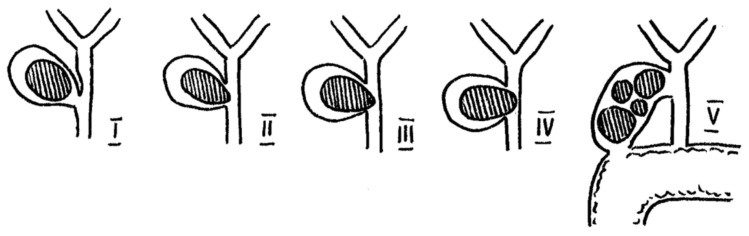
Anatomical sketch of Mirizzi Syndrome according to Csendes and Beltrán (2008).

Two main, widely used classifications for Mirizzi syndrome can be found in original papers. McSherry’s paper proposes a classification based on ERCP findings and distinguished two types of MS. Furthermore, Csendes’ classification determines four types of MS, but it is based on dividing cholecystobiliary communication into three types according to the size of the cholecystocholedochal fistula in comparison to the common bile duct (CBD) [1,16]. A schematic sketch of MS is presented in Figure 1.

### 1.1. Pathophysiology

The current knowledge on the pathophysiology of Mirizzi syndrome states that it is an external compression of the common hepatic duct (CHD) by a gallstone impacted in the gallbladder’s Hartmann’s pouch or the cystic duct [17,18,19]. Previously, however, concepts for explaining the disease were different. Initially, Mirizzi thought that the syndrome he described was functional, rather than just mechanical. He believed that the inflammation in the surrounding structures predisposes a “bile duct sphincter” to contract and cause biliary stenosis. Later, his theory was discarded due to the lack of any sphincter in that area [17]. There were reports of classifying Mirizzi syndrome as the compression of the bile duct by structures other than a gallstone, such as gallbladder cancer or a large, distended, inflammatory gallbladder, but some authors suggest avoiding such confusion and refer to MS as only a stone disease [11]. Isolated extrinsic compression of the CHD is considered to be the first stage of the syndrome. Prolonged, chronic compression caused by the stone on the gallbladder and CHD walls results in inflammation, ulceration and the formation of cholecystobiliary fistulas of different stages of advancement. A cholecystoenteric fistula may also occur in the same way. These complications are recognised as the next stages of the Mirizzi syndrome [5,8,16,17,18]. Beltrán et al. formed a list of nine characteristics of MS distorted anatomy based on previous reports [17]. The authors marked out:

1. Atrophic gallbladder with thick or thin walls;
2. Obliterated cystic duct;
3. Cystic duct—long, parallel to the common bile duct and with low insertion;
4. Cystic duct—short cystic duct with another anatomical variation;
5. Bile duct—partially obstructed due to the external compression or a gallstone eroding from the gallbladder;
6. Distal bile duct with normal thin walls and no distended lumen;
7. Proximal bile duct—dilated with inflamed walls;
8. Abnormal communication between the bile duct and the gallbladder;
9. Fistula between the gallbladder and stomach, duodenum, colon or other structures.

A long-lasting inflammatory process results in the formation of many dense, hard, fibrous adhesions between the gallbladder and the CBD, which are commonly found in patients with MS. Other signs that might suggest Mirizzi syndrome are a thick-walled gallbladder or a contracted gallbladder—especially when a fistula is present. However, when a fistula is absent, the gallbladder can be enlarged and inflamed due to a cystic duct blockage. Operating in the difficult anatomy and adhesions in MS is challenging and the dissection of Calot’s triangle may lead to bile duct injury or bleeding [6,20,21].

### 1.2. Aim of the Review

Despite the advancement of the tools that modern medicine has, Mirizzi Syndrome is still considered a ‘trap’ in cholelithiasis surgery [11]. The diagnosis of this syndrome cannot be solely clinical; thus, other diagnostic measures, such as laboratory tests and imaging, are necessary [16]. Because a large proportion of all MS cases are diagnosed intraoperatively, which increases the risk of diverse complications [17,18], novel diagnostic approaches are greatly needed for surgeons to improve the results of MS management. The aim of this review is to highlight the proposed classifications for Mirizzi Syndrome and to provide an update on modern approaches to the diagnosis of this disease.

## 2. Literature Research

**Figure 2:**
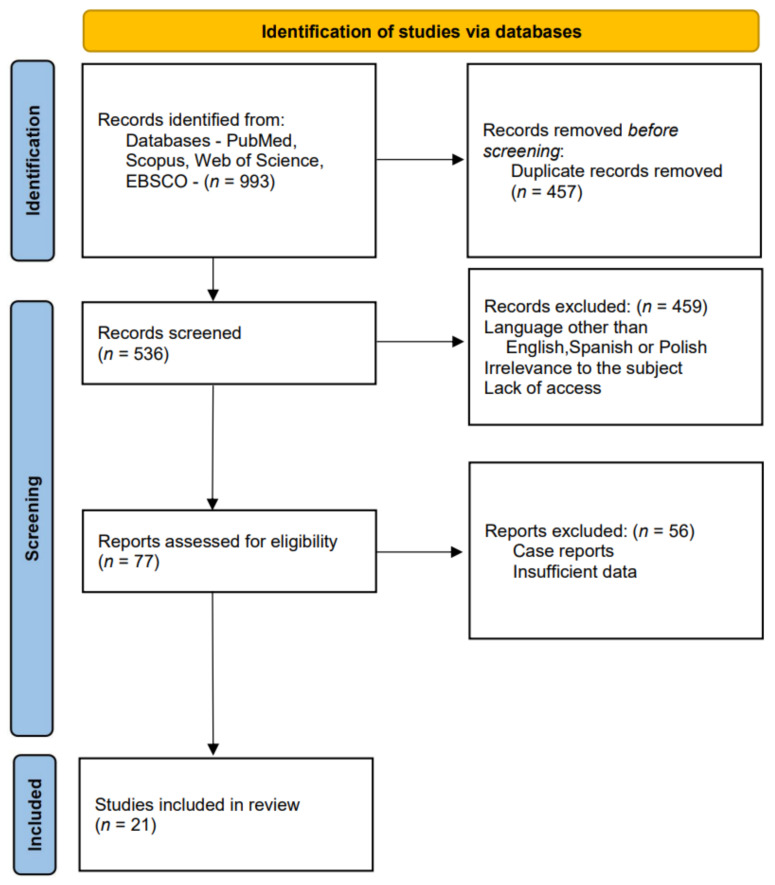
Studies’ identification.

We conducted our literature research in the PubMed, Scopus, Web of Science and EBSCO electronic databases using combinations of the following key words: Mirizzi syndrome, diagnosis. We narrowed the research timeline to the period from 2005 to 2021. The total number of database records comprised 993 articles. After initial selection based on exclusion criteria such as languages other than English, Spanish or Polish, lack of access to a paper, or irrelevance to the subject of Mirizzi syndrome, and after having removed duplicates, we were able to include 77 papers for further evaluation. The final analysis allowed us to select 21 papers for the review (Figure 2).

## 3. Classification

The first classification of Mirizzi syndrome was developed by Corlette et al. in 1975 and they identified two types depending on the degree of cholecystobiliary fistulas [22]. However, the first widely accepted classification, which is still used today, was prepared by McSherry et al. in 1982 based on the ERCP findings. They divided MS into two types, in which type I was an external compression of the bile duct by a gallstone impacted in the neck of the gallbladder and type II was a cholecystobiliary fistula caused by eroded stones [23].

In 1989, Csendes et al. proposed a classification which expanded the one proposed by McSherry. The authors presented four types of the syndrome—type I, which was equal to the McSherry type I; and types II–IV relating to the different stages of the fistula. Type II represents a cholecystobiliary fistula with up to one-third of bile duct wall erosion. Type III consists of a fistula involving two-thirds of the bile duct wall. Finally, type IV refers to the complete destruction of the bile duct and its walls being fused with the gallbladder [24]. This classification remained unchanged for almost two decades, but in 2008 Csendes and Beltrán complemented the previous classification by adding types Va and Vb. Type Va includes an uncomplicated cholecystoenteric fistula, while type Vb represents a cholecystoenteric fistula followed by a gallstone ileus [25].

Meanwhile, in 1997 Nagakawa et al. proposed a different classification based on their own experience, in which types I and II were consistent with the McSherry classification, but type III involved the presence of stones in the confluence of the cystic duct and the common hepatic duct, and type IV was determined as a bile duct stricture without stones, but due to an inflammatory process such as cholecystitis [26].

In 2009 Solis-Caxaj suggested a way to simplify Cesendes and Beltrán’s classification into three types: types I and II were the same as McSherry’s types regarding cholecystoenteric fistulas—IIIa (without gallstone ileus) and IIIb (with gallstone ileus) [27]. Based on this suggestion, Beltrán et al. validated the previous classification in 2012 by implementing Solis-Caxaj types IIIa and IIIb instead of types Va and Vb, but also resigned from the previous types II-IV and simplified them to types IIa (a fistula involving <50% of the bile duct diameter) and IIb (a fistula involving >50% of the bile duct diameter) [17].

In 2017, Payá-Llorente et al. proposed a modified classification based upon Beltrán’s from 2012. The authors in this article make the point that, in their opinion, the presence of a cholecystoenteric fistula should not constitute a type of Mirizzi syndrome, but rather a subtype. Thus they formed a 3 type classification with A, B and C subtypes for each number. Type 1 is extrinsic compression of CHD, types 2 and 3 describe a cholecystobiliary fistula that affects <50% of CBD (2) and >50% of CBD (3). Subtypes A, B and C always correspond to the cholecystoenteric fistula in which A means no fistula, while B and C refer to a fistula with (B) or without (C) gallstone ileus. The researchers also suggested proper schemes of treatment along with their classification, which can be helpful in planning the management of MS [19]. The above classifications have been gathered in Table 1.

Among the articles that were reviewed, one study conducted by Ya Feng Ji et al. compared two classifications—by Csendes and by Nagakawa—for their accuracy in MS diagnosis by computed tomography (CT) and magnetic resonance imaging (MRI). The evaluation proved Nagakawa‘s system to be superior to Csendes’ in terms of diagnostic accuracy [18].

## 4. Symptoms and Laboratory Results

Several symptoms were acknowledged in patients suffering from MS. According to the original papers, the most common symptoms were abdominal pain (incidence 65.7–100%) and jaundice (ranging between 45–87.5%). Other symptoms were nausea and vomiting (31–62%), cholangitis (up to 56%), fever (21–42%) and anorexia (11–29.2%) [6,7,28,29,30,31,32]. Furthermore, Shirah et al. reported that there was a positive Murphy’s sign in 50% of their patients during physical examination [6]. The mean duration of the various symptoms was determined to be between 3 to 24 months [13,19,20], but it is worth mentioning that Prasad et al. noticed that symptoms in patients suffering from uncomplicated gallstone disease lasted half as long as in those with MS [33]. The overall percentage of asymptomatic patients ranged between 3.7% and 17% [28,34].

Several original articles present laboratory test results performed on patients with MS. The most common examinations are white blood cell count (WBC), alanine aminotransferase (ALT), aspartate aminotransferase (AST), alkaline phosphatase (ALP), bilirubin and gamma-glutamyl transpeptidase (GGT).

Leukocytosis was diagnosed in 73.4% of MS patients in the study by Shirah et al. [6]. Ahlawat reported elevated WBC only if acute cholecystitis, cholangitis or pancreatitis occurred along with MS [35]. Articles that present numerological data show that mean WBC levels are generally around the upper limit of normal levels or slightly beyond. A few papers specify the results for different types of MS. According to Payá-Llorente and Erben [19,28], mean WBC levels were moderately lower when a cholecystobiliary fistula was present. On the other hand, Lledó et al. presented contrary data with an inverted trend [31]. 

ALT and AST levels are reported to be generally elevated in 39–98% of tests for ALT and 37–89% for AST [6,29,32,34,35]. According to some of the articles, the mean levels of ALT and AST in MS patients are several times higher than normal and can reach 286 and 263 U/L, respectively. Data describing those parameters in relation to MS type are inconsistent. Erben et al. report a significant decline in AST and ALT levels from over 250 U/L to less than 100 U/L when there is a cholecystobilliary fistula, while Lledó et al. show a gradual growth in the levels of the parameters with the advancement of the fistula, but neither ALT or AST exceed 90 U/L in this study [8,15,28,31].

The results of ALP test are said to be elevated in even 93.8% of patients and its mean levels are reported to be approximately 324–402 U/L, but can be as high as 1236 U/L [15,20,29,32,34,36].

Most authors concur that total bilirubin levels are elevated in MS patients—even in as many as 92.2% of them [6,32,34]. Payá-Llorente as well as Lledó report increasing mean levels of bilirubin with the advancement of the cholecystobiliary fistula. Erben et al., however, present data showing a decline in blilirubin when there is a cholecystobiliary fistula. Interestingly, Payá-Llorente and colleagues report much lower bilirubin in Csendes type V, which may be due to a discharge of the bile straight to the intestines. Generally, the mean bilirubin levels are reported to be between 2–9.9 mg% [8,15,19,20,28,31,36].

The literature is consistent when it comes to GGT levels, which are commonly elevated according to multiple articles. The mean range could be depicted as 204–1018 U/L [15,19,29,34,36].

## 5. Imaging

According to Seah et al., making a preoperative diagnosis of Mirizzi Syndrome is a challenge. Their study was conducted from 2001 to 2012, based on 64 patients who underwent surgery for MS, which has been discovered preoperatively in 48 out of the 64 cases (75%). Although the ultrasonography (US) was initially performed in 53.8% of the cases, it was suggestive in only 4 out of the 35 patients (11.4%). CT findings led to a presumptive diagnosis in 40% of cases (16 out of 40 patients). However, the importance of the CT scan lies in detecting any possible malignancy—CT images confirmed the presence of carcinoma in all three cases (100%), after the postoperative discovery. In this study, the most accurate diagnostic method was MRCP, which was performed on 27 out of 64 patients. The procedure was carried out when a better depiction of the biliary tract was required. Magnetic resonance cholangiopancreatography (MRCP) suggested MS in 24 patients (88.9%). The intraoperative discovery of a fistula occurred in 12 cases, while MRCP images implied the finding preoperatively in 2 patients (7.4%). Although the sensitivity of ERCP was lower (65.9%) compared to MRCP, it provided a diagnosis in 29 out of 44 patients [32].

The diagnosis of Mirizzi Syndrome has been also been analysed by Acquafresca et al. MS occurred in 14 out of 2160 patients who underwent surgery due to biliary lithiasis (0.65%). An US had been performed in every case—it showed a gallbladder affected by gallstones in 71.43% of cases and a dilated intrahepatic bile duct in 92.86% of patients. The authors mention that although the US should be the first method to be used when MS is suspected, a preoperative diagnosis based on US findings was possible in only one case (7.14%). MRCP, which is regarded to be a procedure of high sensitivity and specificity, was carried out on two patients and suggested a preoperative diagnosis of Mirizzi Syndrome. The remaining 11 cases were diagnosed intraoperatively (78.57%) [13].

Xu et al. collected data of 27 patients with MS from the period between 1998 and 2011, when 8697 cholecystectomies were performed. The incidence of MS was 0.31% and the Csendes classification was used to determine the type of MS. All patients underwent ultrasonographic examination and among them 12 (44.4%) were diagnosed correctly. CT was proven to be slightly more effective than US, with four out of eight patients diagnosed with MS. MRCP proved to have better imaging modality, with nearly 80% success in diagnosis. ERCP, however, was decisive, with 100% sensitivity in 17 diagnosed patients [34].

Erben et al. gathered 36 cases of MS from among 21,450 patients undergoing cholecystectomies between 1987 and 2009. The authors used McSherry’s classification to diagnose their patients. The diagnostic modalities which were used were US, CT and ERCP in 27, 24 and 32 patients, respectively. The level of sensitivity registered for these imaging methods were US—48%, CT—42%, ERCP—63% [28].

During the intraductal ultrasonography (IDUS) procedure, the probe is inserted up until it reaches biliary bifurcation and after imagining it is withdrawn in a stepwise manner. The criterion for diagnosing Mirizzi syndrome by IDUS is a “caplike” reflex, with or without an acoustic shadow seen in a ductal structure next to the CBD at the level of obstruction. According to Wehrmann et al., the most meticulous disclosure of stones located in the bile duct was achieved with IDUS (sensitivity 95%, specificity 92%), while MRCP achieved a level of sensitivity of 80% and a specificity of 83%. When we compare endoscopic ultrasonography (EUS)—linear or radial—and intraductal ultrasonography, IDUS is superior to EUS in particular with regard to accuracy, sensitivity and specificity. Additionally EUS lacks the clear anatomical guide (the CBD) that IDUS has by definition. The information provided by IDUS was used to specify an appropriate surgical approach and enabled the surgeons to create direct patient management. It led to a higher rate of minimally invasive surgery being carried out on patients [36].

Kamalesh et al. report 20 cases of MS diagnosed from among 1530 cholecystectomies performed between 2006 and 2013, which is an incidence of 1.4%. US was the method of choice in the initial diagnosis. The authors claim that this method was diagnostically effective in 40% of patients. Other diagnostic methods used for a very few patients (although the study does not give the exact number) were CT and MRCP, which were diagnostically effective in 33% and 100% of cases, respectively. Moreover, the researchers mention that they performed EUS—diagnostic in 63% of cases—and ERCP, which had 72% efficacy. Still, 8 out of 20 patients were diagnosed intraoperatively, which represents 40% of all MS patients in this study [20].

A preoperative diagnosis and laparoscopic treatment was evaluated by Kwon et al. Among 2012 cholecystectomies performed in 1992–2005, they report 24 patients diagnosed with MS—which is an incidence of 1.2%. The diagnostic tools used in this study were ERC and spiral CT after an intravenous infusion cholangiography (IVC-SCT). In general, 20 patients were successfully diagnosed with MS preoperatively. The remaining four patients were suspected of having gallbladder cancer, but postoperatively they were eventually classified as MS. ERCP was diagnostic in 9 out of 13 patients (69%), while IVC-SCT proved to diagnose all 11 patients that were subjected to this method of diagnosis. However, it is worth mentioning that the patients diagnosed by IVC-SCT were all MS type I (McSherry) [37].

The diagnostic possibilities and the individual pros and cons of imagining methods in MS were analysed by Cui et al. They retrospectively investigated 198 patients with Mirizzi Syndrome between 2004 and 2010. US as a widely available and noninvasive tool was the initial diagnostic practice in every case. It revealed gallstones and inflammation within the gallbladder in all patients. Apart from this, other findings, such as shrunken gallbladder walls, dilated CHD and acute cholecystitis with a distended edematous gallbladder, were found in 77.8% patients and this led to the suggestion of MS. In this study, MRCP was performed in 82.3% of cases and it had higher sensitivity for patients with a cholecystobiliary fistula than in those without. For 89 patients (44.9%) it was necessary to perform CT to differentiate MS from a malignant disease. Only 31.3% (62 patients) underwent ERCP due to ambiguous USG and MRCP examinations [8].

In the article by Kumar et al., the authors analysed 169 cases of Mirizzi syndrome managed by surgeons between 1989 and 2011. Only 32% (54/169) were diagnosed preoperatively on the basis of an imaging study. The methods that were available and used from 1989 were US, CT, ERCP and from 2004—MRCP. According to the data, a transabdominal US was conducted in all patients but it led to diagnosis in only 17 (10%) of them. Apart from that, 48 patients (28%) underwent contrast-enhanced CT, as a result of which 5 were successfully diagnosed with MS. ERCP was conducted in 101 (60%) cases, from which MS was identified in 27 of them (27%). Later, 12 patients suspected of having MS went through MRCP and the diagnosis was confirmed in 5 cases. The gender ratio in this group of patients was 107:62 (63:37%) with a preponderance of women [7].

A group of 28 patients with MS was analysed by Payá-Llorente et al. Among 4853 cholecystectomies between 2000 and 2015, the incidence of MS was 0.5%. Csendes classification was used to describe the type of MS, but subsequently patients were classified using a new classification system proposed by the authors. All the patients underwent US examination, which proved to be diagnostically successful in 50% of them. Overall a preoperative diagnosis was made for 19 patients (68%). The authors reported 4 and 2 patients diagnosed by CT and MRCP, respectively, although the exact number of patients subjected to those methods of diagnosis is not given. Similarly, 7 patients underwent ERCP prior to surgery, but its effectiveness in diagnosing MS was not specified. The frequency of intraoperative cholangiography, which was introduced in 24 patients (86%) in this series, is worth mentioning [19].

Testini et al. investigated a group of 18 patients with MS. Although US was performed on every patient at admission, it did not lead to presumptive diagnosis. CT findings could be interpreted as a carcinoma rather than other gallbladder diseases, which is why it was carried out additionally, when MS was an intraoperative discovery in patients with acute abdominal problems. MRCP, considered to be the most accurate method for diagnosing MS, was conducted only when the presence of acute abdominal problems or obstructive jaundice was visible (*n* = 5, 27.8%), mostly because its price makes it an inaccessible imaging method for the vast majority of the patients. Nonetheless, the findings in the procedure suggested a diagnosis of MS. It was necessary to carry out ERCP for five patients who had obstructive jaundice, cholangitis or pancreatitis [38].

Nassar et al. analysed a database of 5740 laparoscopic cholecystectomies performed by a single surgeon between 1992 and 2020. Among these, 58 cases of MS were reported. US was the only diagnostic method in 34 patients (59%)—it revealed dilated bile ducts in the vast majority of cases, but suggested a diagnosis of MS in one patient as a result of the presence of bile duct stones. Although CT scans were carried out on eight patients before referral to the biliary firm and in three cases after the referral, they did not lead to a diagnosis. Nine MRCP were carried out prior to referral and three after, but overall that method only suggested MS in three cases. However, 10 patients underwent ERCP preoperatively (7 before the referral), which led to the diagnosis of MS in only 3 patients. In general, radiological investigations led to a presumptive diagnosis in seven cases (12%). All the remaining cases (*n* = 51, 87.9%) were discovered intraoperatively [10].

Gomez et al. reported a series of patients with Mirizzi syndrome who were managed between 1994 and 2005. US was carried out in 32 of the total 33 patients. Based on US findings such as a gallstone impacted at the neck of the gallbladder or the cystic duct and an associated dilatation of the biliary tract, they managed to successfully diagnose only four cases (12%). According to the authors, additional imaging is often required due to the difficulty in differentiating MS from malignancy in the biliary tract or the gallbladder. In 28 cases (85%) second line investigations following no definitive diagnosis from the US were contrast enhanced CT (*n* = 9), MRCP (*n* = 14) and ERCP (*n* = 11). CT was indicative of MS in three patients and led to a MS diagnosis in two. A diagnosis was achieved with MRCP in 10 patients and with ERCP in 11 patients. In the remaining five patients, MS was identified intraoperatively [30].

Shirah et al. conducted a study of a retrospective cohort database analysis (2003–2012) of patients treated for MS. They divided a total of 64 patients into 3 groups based on clinical features: (1) incidental diagnosis of MS intraoperatively; (2) patients diagnosed by ERCP, who had jaundice; (3) an initial diagnosis by ultrasound. The type of MS was determined according to Csendes classification. US was the diagnostic method of choice performed on all 64 patients, but it was diagnostically effective in only 13 (20%). The authors gave information about 17 cases detected by ERCP and 14 by CT, although the total number of patients who underwent this diagnosis was not presented. An intraoperative diagnosis was made in 53% of cases and interestingly all those patients were classified as MS type I [6].

Prasad et al. analysed connections between MS and gallbladder cancer. There were 133 patients diagnosed with MS out of 4800 cholecystectomies. Preoperative diagnosis was made by cholangiographic findings and was possible in 32% of patients. This means that the intraoperative diagnosis rate was high and reached 68%. Among the MS patients, 7 were simultaneously diagnosed with gallbladder carcinoma (GBC) (5.3%) [33].

During a 6-year period Lledó et al. analysed 35 cases of MS highlighted from a total of 1168 cholecystectomies performed from January 2006 to November 2012 (2.8%). The rate of preoperative diagnosis was 68.5% (24 cases) versus 31.5% intraoperatively. The patients were diagnosed by different imaging methods. All of them underwent a hepatobiliary US (sensitivity 51%), 27 had MRCP, 16 ERCP and CT was performed in 12 cases. An endoscopic sphincterotomy was introduced in patients with confirmed lithiasis (in this study—6 cases). There were no data describing levels of sensitivity in MRCP, ERCP and CT [31].

## 6. Discussion

Mirizzi syndrome is attracting the interest of researchers around the world and this is evidenced by the development of classifications of this disease. As our review shows, the Csendes classification is undoubtedly the most commonly used—13 out of 16 authors referred to it. The remaining three used the McSherry classification (Table 2). The Csendes classification is very precise in describing the advancement of MS, but since it is very difficult to establish the exact degree of bile duct wall destruction preoperatively, its practical use may be limited [21]. Thus recent classifications tend to simplify and rearrange the previous ones in order to propose adequate treatment schemes based upon them. We believe that Payá-Llorente et al. have created the most accurate classification, both pathologically and clinically [19].

The literature confirms the main symptoms of MS, which are upper right quadrant abdominal pain and jaundice. The articles that were reviewed also revealed several laboratory parameters to be abnormal. MS as a biliary disease causes ALT, AST, ALP, GGT and bilirubin levels to be mostly elevated. Due to the inflammatory process, WBC is also generally high. However, the numbers can vary at different stages of the syndrome.

Ultrasonography was the initial diagnostic tool used in a number of studies, but its sensitivity ranged from a few percent up to about 50%. One study, however, reported that US could reveal a suspicion of MS in almost 80% of patients. In our opinion, suspected MS in an US is enough information to guide the next steps in the diagnostic process. Some authors claim that introducing modern US imaging could raise the effectiveness of this method [21].

CT proved to be approximately as sensitive as the US in our review, but it was repeatedly pointed out that its main advantage is differentiating MS from malignant strictures [31,32]. MS is listed as one of the diseases mimicking cholangiocarcinoma, but the fact us that the coexistence of MS and GBC is no less important [33,39,40,41]. Prasad et al. reported that patients with MS and simultaneous GBC were a decade older than and had twice as long a history of symptoms as patients who only had MS alone [33].

MRCP and ERCP compete for the best diagnostic modality. Both showed high levels of sensitivity (Table 2)—in general 63–89% for MRCP and 63–72% for ERCP with singular reports of lower and higher effectiveness. Nevertheless, ERCP is still widely recognised as the gold standard in MS diagnosis, thanks to its high sensitivity and therapeutic options [20,38]. The low availability and high cost of MRCP are its main drawbacks, preventing it from being commonly used [38]. Furthermore, MRCP without a conventional MRI may sometimes struggle to differentiate benign from malignant causes of biliary stricture [36]. Yun et al. report that combining MRCP and CT might increase the number of patients being diagnosed [42].

Some other diagnostic methods emerge in original papers. Authors report EUS as a relatively good tool with a level of sensitivity similar to ERCP ranging from 63–73% [20,36]. Interesting data are delivered by Wehrmann et al. about IDUS, which was reported to be diagnostically accurate in 97% of cases. According to the article, the biggest concerns preventing the widespread use of IDUS are the costs, the length of the procedure and the technical difficulties caused by damage to the IDUS probes. However, the authors mention that the cost of one probe, which can be used several hundred times, is about EUR 3500 euros, and the additional time needed to perform IDUS was 8 min on average [36].

Recently, Tataria et al. published a study that is new in the field of MS management and diagnosis. On the basis of research from various databases and after taking into account clinical, biochemical and radiological parameters presented in MS, the authors developed a scoring system to help predict Mirizzi syndrome preoperatively. The scale consists of 10 parameters gathered in 3 groups: clinical, biochemical and imaging. Each parameter is given 0 or 1 point. In clinical parameters +1 point could be noted for: symptom duration symptoms >24 months; the frequency of abdominal pain >1; and the presence of jaundice. The biochemical results which are given +1 point are: bilirubin level >1.2 mg%; leukocytosis >11,000/mm^3^; and alkaline phosphatase level >150 U/L. The radiological features included in the scale are: the presence of hepatolithiasis/choledocholithiasis; intrahepatic biliary radical dilatation (IHBRD); meniscus sign; and mass at the confluence. The analysis included retrospectively collected data from 96 patients with complicated cholecystitis, who were divided into two groups—the first-without MS and the second with MS. There were 32 patients with MS. The authors further evaluated the patients according to the scoring system they had developed and conducted a statistical analysis which showed that a score of 3 or more out of 10 has a sensitivity of 90.6% and specificity of 78.1% in predicting the Mirizzi syndrome [41].

According to our review, making a preoperative diagnosis is still difficult, but the numbers vary greatly—between 12% and 84%. Many authors underline the importance of a preoperative diagnosis of MS to avoid exposing surgeons to difficult operating conditions and therefore to limit complications by choosing the right approach [43,44,45,46]. Along with a precise diagnosis, proper treatment must follow. Open cholecystectomy is in general accepted as the procedure of choice, yet some studies recommend a laparoscopic approach, which is said to be safe, especially when there is no cholecystobiliary fistula [20,43]. However, it is crucial that MS is diagnosed preoperatively when planning laparoscopic treatment. Research shows a high conversion rate when the diagnosis in not made prior to the surgery and this increases with the advancement of the disease [43,47].

Endoscopic interventions are also reported to be good options for the management of MS. Introducing nasogallbladder drainage, stenting or even laser lithotripsy can complement surgical treatment and decrease the number of complications [20,46,48].

Several diagnostic and therapeutic algorithms have been proposed for MS [6,37,38,49]. There is as yet no conventional agreement as to which classification or what kind of approach should be administered to treat a specific type of the disease. Therefore, we believe that a wider consensus in this matter is needed to unify standards. However, to allow for future studies, researchers should pay more attention to providing more detailed numbers when it comes to their patients, as some papers currently lack specific information. Due to the rarity of MS, a multicentred study would be necessary in order to obtain sufficient data from which to draw unambiguous conclusions. We strongly encourage future researchers to use the classification proposed by Payá-Llorente et al. and the scoring system provided by Tataria et al. to verify their efficacy for practical use, as they may be promising steps forward in MS management.

## Figures and Tables

**Table 1:** Classifications of Mirizzi Syndrome.

Authors	McSherry et al.-1982	Csendes et al.-1989 and Complemented in 2008	Beltrán et al.-2012	Payá-Llorente et al.-2017	Nagakawa et al.-1997
**Classification**	Type I—external compression of the bile duct	Type I—external compression of the bile duct	Type I—external compression of the bile duct	Type 1—external compression of the bile duct	Type I—external compression of the bile duct
	Type II—cholecystobiliary fistula	Type II—cholecystobiliary fistula—up to 1/3 of the bile duct wall erosion	Type IIa—cholecystobiliary fistula involving <50% of the bile duct diameter	Type 2—cholecystobiliary fistula involving <50% of the bile duct diameter	Type II—cholecystobiliary fistula
		Type III—cholecystobiliary fistula—up to 2/3 of the bile duct wall erosion			
		Type IV—cholecystobiliary fistula—complete destruction of the bile duct wall and fusion with gallbladder	Type IIb—cholecystobiliary fistula involving >50% of the bile duct diameter	Type 3—cholecystobiliary fistula involving >50% of the bile duct diameter	
		Type Va—cholecystoenteric fistula	Type IIIa—cholecystoenteric fistula	Subtypes describing cholecystoenteric fistula: A-no fistula/B-fistula without gallstone ileus/C-fistula with gallstone ileus	Type III—gallstones in the cystic duct and common hepatic duct confluence
		Type Vb—cholecystoenteric fistula with gallstone ileus	Type IIIb—cholecystoenteric fistula with gallstone ileus		Type IV—stricture without stones (e.g., due to cholecystitis)

**Table 2:** Review studies of case series, arranged in order of concurrence in the ‘Imaging’ section of the article.

	Authors and the Year of Publication	Period of Studied Patients	Analysed Patients	The Patients with MS	Men/Women	Classification	Patients Diagnosed Preoperatively	Patients Diagnosed with:					Patients Diagnosed Intraoperatively or Postoperatively
								US	CT	MRCP	ERCP	Other	
1	Seah et al., 2018 [32]	November 2001–June 2012	10,176	64	27/37	Csendes	48 (75%)	4/35 (11.4%)	16/40 (40%)	24/27 (88.9%)	29/44 (65.9%)	-	16 (25%)
2	Acquafresca et al., 2014 [13]	July 2007–June 2013	2160	14	6/8	Csendes	3 (21.43%)	1/14 (7.14%)	-	2/14 (14.3%)	-	-	11 (78.57%)
3	Xu et al., 2013 [34]	January 1988–November 2011	8697	27	8/19	Csendes	-	12/27 (44.4%)	4/8 (50%)	7/9 (77.8%)	17/17 (100%)	-	-
4	Erben et al., 2011 [28]	January 1987–December 2009	21,450	36	19/17	McSherry	-	13/27 (48%)	10/24 (42%)	-	20/32 (63%)	-	-
5	Wehrmann et al., 2006 [36]	January 2002–December 2004	2089	30	-	-	-	-	1 (3%)	12/19 (63%)	-	IDUS 29/30 (97%); EUS 11/15 (73%)	-
6	Kamalesh et al., 2015 [20]	January 2006–August 2013	1530	20	11/9	Csendes	12 (60%)	- (40%)	- (33%)	- (100%)	13/18 (72%)	EUS - (63%)	8 (40%)
7	Kwon et al., 2007 [37]	April 1992–December 2005	2012	24	15/9	McSherry	20 (83%)	-	-	-	9/13 (69%)	IVC-SCT 11/11 (100%)	4 (17%)
8	Cui et al., 2012 [8]	January 2004–January 2010	29,875	198	88/110	Csendes	-	Suggested in 154/198 (77.8%)	Performed in 89 to differentiate from malignant disease	Suggested in 163	-	-	-
9	Kumar et al., 2016 [7]	1989–2011	169	169	62/107	Csendes	54 (32%)	17 (10%)	suspected in 5	5	27	-	115 (68%)
10	Payá-Llorente et al., 2017 [19]	January 2000–October 2015	4853	28	7/21	Csendes	19	14 (50%)	4	2	-	-	-
11	Testini et al., 2016 [38]	March 2006–February 2016	919	18	6/12	Csendes	5 (27.8%)	-	-	5/5 (100%)	-	-	13 (72.2%)
12	Nassar et al., 2020 [10]	February 1992–February 2020	5740	58	20/38	Csendes	7 (12%)	1/34 (2.9%)	0/11	3/12 (25%)	3/10 (30%)	-	51 (88%)
13	Gomez et al., 2006 [30]	August 1994–August 2005	33	33	15/18	McSherry	28 (84.4%)	4	3	10	11	-	5 (15.2%)
14	Shirah et al., 2017 [6]	January 2003–December 2012	-	64	15/49	Csendes	30 (46.9%)	13/64 (20.3%)	14	-	17	-	34 (53.1%)
15	Prasad et al., 2006 [33]	1989–2003	4800	133	42/91	Csendes	- (32%)	-	-	-	-	-	- (68%)
16	Lledó et al., 2014 [31]	January 2006–November 2012	1168	35	-	Csendes	24 (68.5%)	- (51%)	-	- (76%)	-	-	11 (31.5%)
17	Waisberg et al., 2005 [29]	November 1997–June 2003	8	8	4/4	Csendes	1 (12.5%)	One patient diagnosed by combining US, CT and ERCP					7 (87.5%)

## Data Availability

Data sharing not applicable.
